# Trials of Pharmacological Interventions for Tourette Syndrome: A Systematic Review

**DOI:** 10.3233/BEN-2012-120269

**Published:** 2013

**Authors:** Karen Waldon, Jonathan Hill, Cristiano Termine, Umberto Balottin, Andrea Eugenio Cavanna

**Affiliations:** ^1^The Michael Trimble Neuropsychiatry Research GroupDepartment of NeuropsychiatryBSMHFT and University of BirminghamBirminghamUK; ^2^Child Neuropsychiatry UnitDepartment of Clinical and Experimental MedicineUniversity of InsubriaVareseItaly; ^3^Child Neuropsychiatry UnitIRCCS 'C. Mondino Institute of Neurology' FoundationUniversity of PaviaPaviaItaly; ^4^Department of Motor Neuroscience and Movement DisordersInstitute of Neurology and University College LondonLondonUK

**Keywords:** Drug therapy, movement disorder, pharmacological treatment, randomised controlled trial, tics, Tourette Syndrome

## Abstract

*Introduction:* Gilles de la Tourette Syndrome (GTS) is a childhood-onset hyperkinetic movement disorder defined by the chronic presence of multiple motor tics and at least one vocal tic and often complicated by co-morbid behavioural problems. The pharmacological treatment of GTS focuses on the modulation of monoaminergic pathways within the cortico-striato-thalamo-cortical circuitry. This paper aims to evaluate the efficacy and safety profiles of pharmacological agents used in the treatment of tics in patients with GTS, in order to provide clinicians with an evidence-based rationale for the pharmacological treatment in GTS.

*Method:* In order to ascertain the best level of evidence, we conducted a systematic literature review to identify double-blind randomised controlled trials of medications in GTS populations.

*Results:* We identified a large number of pharmacological agents as potentially effective in improving tic symptoms. The alpha-2 agonist Clonidine is amongst the agents with the most favourable efficacy-versus-adverse events ratio, especially in patients with co-morbid attention-deficit hyperactivity disorder, although effect sizes vary evidence-based studies.

*Discussion:* Our results are in line with the findings of uncontrolled open-label studies. However, most trials have low statistical power due to the small sample sizes, and newer agents, such as Aripiprazole, have not been formally tested in double-blind randomised controlled trials. Further research should focus on better outcome measures, including Quality of Life instruments.

## 1. Introduction

Gilles de la Tourette Syndrome (GTS) is a childhood-onset neurological disorder [1] characterised by repetitive, stereotypical involuntary movements and vocalisations known as tics [2], which vary in severity over weeks to months [3]. GTS is often associated with behavioural problems, such as attention deficit hyperactivity disorder (ADHD) and obsessive-compulsive disorder (OCD) [4,5]. It is estimated that GTS affects 1–8 per 1000 children [6] worldwide [7], in different degrees of severity [7], however due to misdiagnosis in reality prevalence may be even higher.

Current treatment options for GTS include behavioural therapy [8], medication [9] and neurosurgery [10]. Examples of psychobehavioural interventions are exposure and response prevention [11], massed practice and habit reversal training [11], as well as relaxation therapy [11], biofeedback [11] and acupuncture [12].

ISSN 0953-4180/13/$27.50 © 2013 – IOS Press and the authors. All rights reserved

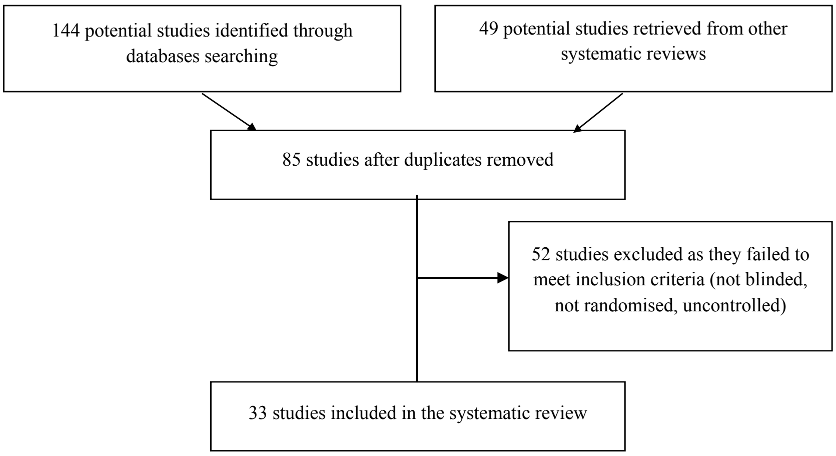

Fig. 1. Flow chart summarising the study selection process.

Medications include typical antipsychotics (neuroleptics) [13,14], atypical antipsychotics [15–17], alpha-adrenergic agonists [18–20], along with other pharmacological classes [21–27].

Historically, neuroleptics such as haloperidol and pimozide have been the mainstay of treatment [4,28], however due to their metabolic and extrapyramidal side effects [28–30] focus has more recently shifted to other agents, such as atypical antipsychotics [15,16,31, 32], which have variable D2 receptor blocking properties and 5-HT receptor blocking properties [33]. These however can also cause metabolic side effects, such as weight gain [15,29,31], which increases risk of cardiovascular disease and type 2 diabetes [27,34].

As a relatively common condition, GTS can cause disruption at school and embarrassment for the child [35] subsequently impacting on their education [20] in addition to difficulties with social interactions [20]. Improving pharmacological control over tics could alleviate these problems [9] but may cause adverse effects of their own [33], outweighing the benefits.

In this paper, we set out to systematically review the existing evidence on medications used to treat GTS. Our aim was to evaluate these agents' efficacy (reduction in tic frequency and severity as well as overall impairment), weighed up against risk of adverse effects, in order to inform clinical practice about the evidence-based rationale for the choice of the most appropriate treatment and ultimately provide better care for this patient population.

## 2. Methods

We conducted a systematic review of the available literature, with focus on randomised, controlled, double-blind studies comparing pharmacological therapies to placebo for the treatment of GTS. Both parallel group and cross-over study designs were included.

Our electronic searches were conducted on the databases Medline and PsycINFO. The first search cross-referenced "Tourette" and its derivations with drug names, as MeSH headings and as text words in the title and abstract fields. Both search terms were required to be major descriptors. The same cross-referenced search was carried out with "tic" in place of "Tourette". Type of article was restricted to original trials on human subjects, published in peer-reviewed journals. Only articles published in the English language since 1960 were included. Since GTS is a disorder which first presents in childhood, we included both children and adult studies.

Relevant journals were manually searched for additional articles using indexes or online search engines. These journals included Neurology, American Journal of Psychiatry, Journal of Neuropsychiatry and Clinical Neurosciences, Movement Disorders, Annals of Neurology, Brain, Journal of Neurology, Neurosurgery and Psychiatry and Journal of Psychosomatic Research. Finally, the reference lists of relevant articles were manually searched for related studies.

The steps of our search methodology are summarised in Fig. 1.

## 3. Results

Thirty-three studies (17 parallel trials and 16 crossover trials) involving a total of 1,385 patients fulfilled our inclusion criteria and were included in the

Table 1
Double-blind randomised controlled studies investigating the efficacy of antipsychotic medications for tic management in Gilles de la Tourette syndrome

| Medication | Author | Year | Design | N | Age range (years) | Treatment duration (weeks) | Effect on tics | Advantages | Disadvantages |
|---|---|---|---|---|---|---|---|---|---|
| *Typical antipsychotics* | | | | | | | | | |
| Haloperidol | | | | | | | | | |
| | Silver et al. | 2001 | Parallel | 70 | | 6.5 | + | | SE: Some cases of nausea and vomiting |
| | Ross and Moldofsky | 1978 | Parallel | 9 | | 80 | + | | |
| | Sallee et al. | 1996 | Crossover | 26 | 7–13 | 6 | + | | |
| | Sallee et al. | 1997 | Crossover | 22 | 7–16 | 6 | − | | SE: 3-fold higher frequency of serious SE and greater ESE than Pimozide |
| | Shapiro et al. | 1989 | Parallel | 57 | | | + | | SE common |
| Pimozide | Bruggeman et al. | 2001 | Parallel | 41 | 11–65 | 7 | + | ESE rare | SE: Sedation and Weight Gain |
| | Gilbert et al. | 2004 | Crossover | 13 | 7–17 | 4 | + | No associated ECG changes Less weight gain than Risperidone | |
| | Ross and Moldofsky | 1978 | Parallel | 9 | | 80 | + | Less lethargy than Haloperidol | |
| | Sallee et al. | 1996 | Crossover | 26 | 7–13 | 6 | + | | |
| | Sallee et al. | 1997 | Crossover | 22 | 7–16 | 6 | + | Less SE than Haloperidol | |
| | Shapiro et al. | 1989 | Parallel | 57 | | | + | | SE: Prolonged QTc interval |
| | Shapiro et al. | 1984 | Crossover | 20 | | 6 | + | | |
| | Onofrj et al. | 2000 | Crosover | 4 | 19–40 | 52 | + | | SE: Sedation and minor motor SE |
| *Atypical antipsychotics* | | | | | | | | | |
| Risperidone | | | | | | | | | |
| | Bruggeman et al. | 2001 | Parallel | 41 | 11–65 | 7 | + | ESE rare | SE: Sedation and Weight Gain |
| | Gilbert et al. | 2004 | Crossover | 13 | 7–17 | 4 | + | More effective than Pimozide | SE: Greater weight gain than Pimozide |
| | Dion et al. | 2002 | Parallel | 48 | | 8 | + | No increase in OCS | SE: Fatigue, somnolence, hypokinesia, tremor |
| | Scahill et al. | 2003 | Parallel | 34 | 6–62 | 8 | + | No ESE | SE: Acute social phobia, weight gain |
| | Gaffney et al. | 2002 | Parallel | 21 | 7–17 | 8 | + | No ESE | SE: Sedation |
| Olanzapine | Onofrj et al. | 2000 | Crossover | 4 | 19–40 | 52 | + | More effective than Pimozide | SE: Minor sedation |

Abbreviations: N = number of subjects in the study; SE = Side Effects; ESE; Extrapyramidal Side Effects; OCS = Obsessive Compulsive symptoms; TSSG = Tourette's Syndrome Study Group.

present review. Table 1 summarises the double-blind randomised controlled studies investigating the efficacy of typical [14,30] and atypical antipsychotic medications [15–17,31,32] for tic management in GTS, whilst Table 2 focuses on the non-antipsychotic medications: alpha2 adrenergic agonists [18–20,36], anticonvulsants [23], dopamine agonists [24,36,37], GABA-modulating agents [21], substituted benzamides [5,38], anticholinergic agents [26], cannabinoids [27], anti-emetics [25,39], 5HT3 antagonists [25], Selective Serotonin Reuptake Inhibitors (SSRIs) [5,40], Selective Norepinephrine Reuptake Inhibitors (SNRIs) [41], tricyclic antidepressants [42], MAO inhibitors and antiandrogens [43,44].

Table 2
Double-blind randomised controlled studies investigating the efficacy of non-antipsychotic medications for tic management in Gilles de la Tourette syndrome

| Medication | Author | Year | Design | N | Age range (years) | Treatment duration (weeks) | Effect on tics | Advantages | Disadvantages |
|---|---|---|---|---|---|---|---|---|---|
| *Alpha adrenergic agonists* | | | | | | | | | |
| Clonidine | Goetz et al. | 1987 | Crossover | 30 | | | – | | |
| | Singer et al. | 1995 | Crossover | 34 | 7–13 | 6 | – | Helped with ADHD symptoms | SE: Sedation |
| | Hedderick et al. | 2009 | Crossover | 10 | 8–27 | 6 | + | | SE: Sedation |
| | Gaffney et al. | 2002 | Parallel | 21 | 7–17 | 8 | + | No ESE | SE: Sedation |
| | Du et al. | 2008 | Parallel | 437 | 6–18 | 4 | + | No SE | |
| | TSSG | 2002 | Parallel | 103 | 7–14 | 16 | + | Helped with ADHD symptoms | SE: Sedation |
| Guanfacine | Scahill et al. | 2001 | Parallel | 34 | 6–14 | 8 | + | Helped with ADHD symptoms | SE: Sedation |
| *Anticonvulsants* | | | | | | | | | |
| Topiramate | Jankovic | 2009 | Parallel | 20 | 7–65 | | + | No observed adverse SE. | |
| Levetiracetam | Hedderick et al. | 2009 | Crossover | 10 | 8–27 | 6 | – | | SE: Irritability |
| | Smith-Hicks et al. | 2007 | Crossover | 22 | 9–14 | 4 | – | | |
| *Dopamine agonists* | | | | | | | | | |
| Pergolide | Gilbert et al. | 2000 | Crossover | 24 | 7–17 | 6 | + | Well tolerated and no serious SE. | |
| | Gilbert et al. | 2003 | Parallel | 57 | 7–17 | | + | Well tolerated, helps with ADHD symptoms | |
| Talipexole | Goetz et al. | 1994 | Parallel | 13 | | | – | | SE: Sedation, dizziness |
| *GABA modulating agents* | | | | | | | | | |
| Baclofen | Singer et al. | 2001 | Crossover | 9 | | 4 | + | No major side effects | YGTSS scores showed reduction in impairment scores rather than decrease in tics |
| *Substituted benzamides* | | | | | | | | | |
| Sulpiride | George et al. | 1993 | Crossover | 11 | | | + | | |
| Tiapride | Eggers et al. | 1988 | Crossover | 17 | | | + | Minimal SE: hyperprolactinaemia restricted to duration of therapy | |
| *Acetylcholinergic agents* | | | | | | | | | |
| Nicotine | Silver et al. | 2001 | Parallel | 70 | | 2.5 | + | Combination with haloperidol superior to haloperidol only | SE: Nausea and Vomiting common |
| Mecamylamine | Silver, Sheehan et al. | 2001 | Parallel | 38 | 8–17 | 8 | – | Reduction in sudden mood changes and depression | |
| *Cannabinoids* | | | | | | | | | |
| Delta-9-tetrahydrocannabinol | Muller-Vahl et al. | 2003 | Parallel | 17 | | 6 | + | No serious adverse SE | |
| *Anti-emetics* | | | | | | | | | |
| Metoclopramide | Nicholson et al. | 2005 | Parallel | 27 | 9–14 | 8 | + | Well tolerated, no significant cardiac changes. Small increase in serum Prolactin | Further trials needed in younger patients |

Table 2, continued

| Medication | Author | Year | Design | N | Age range (years) | Treatment duration (weeks) | Effect on tics | Advantages | Disadvantages |
|---|---|---|---|---|---|---|---|---|---|
| *5HT3 Antagonists* | | | | | | | | | |
| Ondansetron | Toren et al. | 2005 | Parallel | 30 | 12–46 | 3 | − | | SE: Abdominal pain |
| *SSRIs* | | | | | | | | | |
| Fluoxetine | Scahill et al. | 1997 | Crossover | 14 | 8-33 | 8 | − | Reduction in OCS | SE: Transient behavioural activation |
| *SNRIs* | | | | | | | | | |
| Atomoxetine | Allen et al. | 2005 | Parallel | 148 | 7–17 | 18 | + | Useful for ADHD symptoms | SE: Nausea, decreased appetite |
| *Tricyclic anti-depressants* | | | | | | | | | |
| Desipramine | Singer et al. | 1995 | Crossover | 34 | 7–13 | 6 | − | Helped with ADHD symptoms | |
| Fluvoxamine | George et al. | 1993 | Crossover | 11 | | | − | | |
| *MAO inhibitors* | | | | | | | | | |
| Deprenyl | Feigin et al. | 1996 | Crossover | 15 | 7–16 | 8 | + | No SE | |
| *Antiandrogens* | | | | | | | | | |
| Flutamide | Peterson et al. | 1998 | Crossover | 13 | | 3 | +/− (reduction in motor but not phonic tics) | Modest improvement in OCS | Effect is short-lived |

−+ = Positive effect on tic severity; − = Evidence of poor treatment response
Abbreviations: N = number of subjects in the study; SE = Side Effects; ESE; Extrapyramidal Side Effects; OCS = Obsessive Compulsive symptoms; TSSG = Tourette's Syndrome Study Group.

With regards to outcome measures, the majority of trials used Clinical Global Impression (CGI) scale [19,45] and the Yale Global Tic Severity Scale (YGTSS) [45] as the main outcome measure [21,23, 24,27,46–48]. The YGTSS is the total of 2 subscale scores; total tic score (TTS) which rates tic severity from 0–5 and tic impairment score (TIS) which represents how GTS impacts for example family life and self-esteem [23]. The CGI scale rates how much GTS affects a patient's life [23]. Other measures include the Tourette's Disorder Scale-Clinician Rated (TODS-CR) [26] and video tapings used to assess tic frequency by clinicians [36,38,49].

## 4. Discussion

In this paper, we reviewed the existing evidence from randomised double-blind controlled trials investigating the pharmacological treatment of tics in GTS. We critically evaluated efficacy in terms of reduction in tic severity and presence of adverse effects, with the aim to inform clinical practice regarding the choice of pharmacotherapy in GTS.

Antipsychotic medications have long been used for the treatment of tics in GTS [30]. Consistent with early findings, typical antipsychotics Haloperidol and Pimozide demonstrated significant efficacy (reduction in tic severity) [13,14,16,28,30,31,50], however they were also characterised by common side effects, such as sedation [17], weight gain, depression [16], extrapyramidal symptoms [17,30] (especially Haloperidol [28,30]) and risk of QTc prolongation [14,51] (especially Pimozide [14]). According to the available evidence, these agents should not be recommended as first line medications, due to the high rate of adverse effects [28,29]. Among the newer antipsychotics, the use of Risperidone is arguably supported by the best evidence for efficacy [15,16,28,31,32,52,53]. However, weight gain [15,31] and sedation [53] have been reported as common side effects [32], followed by hyperprolactinaemia and risk of extrapyramidal symptoms [16, 32]. The available evidence suggests that Risperidone may not be suitable for overweight patients [28,31], whilst it can prove beneficial for patients with comorbid OCD [16,53] as augmentation therapy to SSRIs for treatment-refractory obsessive-compulsive symptoms. The same is true for substituted benzamides sulpiride [5] and tiapride [5,54–58], although reports of tardive dyskinesia following use of sulpiride use warrant caution [59]. Little evidence was available for new generation antipsychotic medications, such as D2 partial agonist Aripiprazole, which has been recently shown to have positive efficacy and tolerability profiles in a series of case reports and open label studies [51, 60,61].

The centrally acting alpha 2-adrenergic agonist Clonidine [18] has been shown to be a potentially effective and safe option for the first-line treatment of tics in GTS, although effect sizes showed a degree of variability across different studies [3,20,47,53,54,62]. Several studies also reported a significant improvement for patients with comorbid ADHD in impulsive-hyperactive symptoms [19] as well as tic symptoms with both Clonidine [42] and Guanfacine [19]. Among other agents, the newer antiepileptic drugs deserve attention in consideration of the number of trials carried out in GTS populations in recent years. In particular, Topiramate was found to be an effective medication for tic reduction [4], especially in patients suffering from obesity or migraine. However, previous research also reported relatively frequent side effects associated with its use, including concentration difficulties, confusion, ataxia and diplopia [63–65]. The current evidence for Levetiracetam is somewhat conflicting, with a number of studies showing efficacy [66–69], and others showing Levetiracetam being less effective than Clonidine and with no significant improvement in tic severity [23,47]. Moreover, adverse reactions to this medication include irritability, hyperkinesias and insomnia [47].

The reviewed literature presents a number of limitations, which might have a significant impact on the quality of the results we obtained. One main limitation of the trials reviewed in this paper is the high rate of patients lost at follow-up [26], which can be as high as 55% [5], thus reducing the reliability of the results. However ad hoc analysis showed few differences between whole group and group minus drop-outs in endpoint [3,5]. The majority of patients were recruited from specialist clinics; therefore the sample may not be representative of the general population because of the possibility of referral bias. Some of the studies identified were rather small in terms of numbers of participants [17,18,36,40,47]. Small sample sizes mean a relatively small effect in a few patients can result in large treatment effects, which can reduce the reliability of the results. Some studies excluded patients with co-morbid OCD and ADHD, thus reducing the sample size and making the sample less representative. On the other hand, the presence of behavioural co-morbidities can influence the study findings, if not adequately accounted for. Some studies did not provide any information on whether participants had ceased medications (or had a wash-out period) before starting the study, thus leading to potential drug-drug interactions as additional confounding factors [21]. Likewise, the method of randomisation was not reported in all trials. Despite randomisation, there were baseline differences between groups, which in some studies were accounted for using analysis of covariance [25]. Although the results of some studies showed that these differences did not affect the results [37,46], more severe tics at baseline can lead to numerically greater improvements than milder tics [21]. Studies also varied in their approach to determine treatment efficacy, sometimes making it difficult to compare and standardise their results. For example, certain outcome measures such as clinician-assessed video recordings [18,36,49] increase the risk of measurement error and bias. However, most trials were assessed by multiple clinicians and few discrepancies were found between their ratings, indicating a good degree of reliability. Although a number of trials reported a statistical significance in ratings of tic severity, clinical significance was not always apparent. Finally, a mixture of crossover and parallel studies were reviewed, which can have caused some inconsistency in the interpretation of the findings.

Future studies investigating the pharmacological treatment of GTS should be conducted with focus of newly developed, promising agents belonging to pharmaceutical classes with proven efficacy [39], such as the newer antipsychotic Aripiprazole [51,60,61]. With regards to known treatments, further randomised placebo-controlled double-blinded studies are needed with greater sample sizes to increase statistical power, and lower loss to follow up. These studies should ideally use multidimensional outcome measures, such as Quality of Life (QoL) ratings [70], alongside tic severity ratings. Recent research has shown that there is not always a direct correlation between tic severity and QoL, implicating the usefulness of QoL measures in treatment trials. Other primary outcome measures should include ratings of symptoms of co-morbid ADHD and OCD, to address the holistic treatment of patients with GTS. Intention-to-treat analysis should be performed to monitor adverse effects. Finally, samples should also be recruited from different community and clinic populations (e.g. by advertisements in mainstream schools, as well as special education schools and specialist clinics).

## 5. Conclusion

The aim of this paper was to inform clinicians about the appropriate pharmacological management of patients with GTS based on the available evidence. We conducted a systematic literature review focusing on high-quality randomised placebo-controlled trials by using strict search criteria. A number of pharmacological agents were found to be potentially effective in improving tic severity. However some medications (e.g. typical antipsychotics) were characterised by a less favourable profile due to the presence of significant side-effects [46], whilst other agents (e.g. atypical antipsychotics; alpha-2 adrenergic agonists, especially Clonidine [42,71]) showed added benefits of treating co-morbid behavioural symptoms [19,40,41, 43,48]. Caution should be used to avoid the risk of misinterpreting the findings of these studies, as for instance effect sizes show considerable variation in the case of Clonidine. Moreover, the lack of high-level evidence trials makes it difficult to derive conclusions about other promising agents, such as the D2 partial agonist Aripiprazole [51,60,61]. In consideration of the major limitations of the currently available literature (most studies have been conducted on small samples and their results have not been robustly replicated), further research is needed to establish an evidence-based rationale for the choice of first- and second-line pharmacological options for the treatment of tics in patients with GTS.

## Acknowledgements

The authors are grateful to Tourettes Action UK for their continuing support.
